# Facile Preparation of Reduction-Responsive Micelles Based on Biodegradable Amphiphilic Polyurethane with Disulfide Bonds in the Backbone

**DOI:** 10.3390/polym11020262

**Published:** 2019-02-04

**Authors:** Peng Zhang, Jiaying Hu, Leran Bu, Hena Zhang, Baixiang Du, Caihong Zhu, Yuling Li

**Affiliations:** 1 School of Chemistry & Materials Science, Jiangsu Key Laboratory of Green Synthetic Chemistry for Functional Materials, Jiangsu Normal University, Xuzhou 221116, China; zhangpeng@jsnu.edu.cn (P.Z.); hjy960920@163.com (J.H.); b13585393294@163.com (L.B.); zhn8561787@163.com (H.Z.); furan@163.com (B.D.); 2 School of Life Science, Jiangsu Normal University, Xuzhou 221116, China; 3 Orthopaedic Institute, Medical College, Soochow University, Suzhou 215007, China

**Keywords:** reduction-responsive, micelles, polyurethane, triggered release

## Abstract

In this paper, we synthesized a biodegradable amphiphilic polymer of polyurethane-polyethylene glycol with disulfide bonds in the main chain (PEG-PU(SS)-PEG). DLS and SEM showed that the polymer could self-assemble into micelles in aqueous solution and could be used to load the hydrophobic anticancer drug DOX. Intriguingly, drug release in vitro indicated that DOX-loaded PEG-PU(SS)-PEG micelles had good stability under the extracellular physiological environment, but the disulfide bonds broke rapidly and DOX was released quickly under the intracellular reducing conditions. CCK-8 assays showed that DOX-loaded PEG-PU(SS)-PEG micelles had a high in vitro antitumor activity in C6 cells, whereas blank PEG-PU(SS)-PEG micelles were nontoxic to C6 cells. It was also found that there was strong and persistent accumulation of DOX-loaded PEG-PU(SS)-PEG as compared with PEG-PU-PEG both by the cell internalization tests and the flow cytometry measurements. Hence, PEG-PU(SS)-PEG micelles will have a potential use for clinical treatment of cancer in the future.

## 1. Introduction

In recent years, the early detection and effective treatment of cancer still represent a huge challenge due to the disease’s complexity, diversity, and dynamics [1,2]. So far, there is no complete effective solution to the treatment of cancer. Traditional methods of treating cancer include surgical resection, radiotherapy, and chemotherapy, among which chemotherapy is the most common method [3]. However, intravenous administration of many highly effective chemotherapeutic agents is often hampered by their low solubility in aqueous environments [4]. Conventional anticancer chemotherapeutic drugs currently on the market face severe systemic side effects, multidrug resistance (MDR), and deadly metastasis of cancer cells. To address these limitations of chemotherapeutic agents, many drug delivery systems (DDSs) have been studied in order to deliver drugs to tumor sites without harming healthy tissues and organs [5,6,7]. In this case, polymeric micelles have shown great advantages and potential as delivery vehicles for chemotherapeutic drugs, overcoming the problems caused by conventional free drugs, such as poor solubility, poor stability, fast clearance, poor selectivity, and adverse effects on healthy cells, among others [8]. Nanomicelles have good advantages, including improving the solubility of insoluble drugs, being more stable in the body, aiming at the release of anticancer drugs, and prolonging blood circulation time, and they have been applied to clinical treatments [9,10]. Usually, amphiphilic block copolymers undergo microphase separation in aqueous solution to assemble into micelles due to their hydrophilic and hydrophobic interactions. These micelles usually have core–shell structures. The hydrophobic segment bound to the hydrophobic drug constitutes the micellar core, and the hydrophilic segment that increases the stability of the micelle forms the outer shell of the micelles [11]. Poly(ethylene glycol) (PEG), a hydrophilic and nonionic polymer, is the most popularly used hydrophilic segment [12]. Based on its biocompatibility, low dispersity, and limited recognition by the immune system, PEG is widely used to prolong blood circulation time, reduce immunogenicity and toxicity, and optimize protein activities [13,14].

Currently, polyurethanes (PUs) have been identified as one of the most widely available materials for a wide range of biomedical applications. Due to their excellent physical properties and good biocompatibility, they are widely used in clinical applications, ranging from medical devices such as catheters and pacemakers to drug-care hydrogel and nanoparticles [15,16]. Biodegradable PUs are usually prepared via polyaddition reaction from biodegradable polyesters diols and diisocyanates, such as L-lysine diisocyanates (LDI) [17]. PUs have the advantages of easy synthesis, easy modification, and easy introduction of target molecules and ligands, making them a promising carrier for the next generation of nanotherapeutic drugs [18]. Therefore, many block copolymer micelles prepared from PUs are now used in lipophilic drugs and drug delivery systems (DDSs) [19,20]. In 2012, Zhou et al. synthesized biodegradable PU material PCLHxPU by reacting polyethylene glycol-polycaprolactone (PEG-PCL-OH) with different diisocyanates such as LDI, 4,4’-dicyclohexylmethane diisocyanate (HMDI), or isophorone diisocyanate (IPDI) [21]. The PU material was used as a nanocarrier for drug delivery. Cell culture data showed that PU nanoparticles were friendlier to HEK 293 cells [22]. However, drug release data showed that the cumulative release of PU nanoparticles for six days was only 40% of the total dose under normal physiological conditions, indicating that the PU nanoparticles had good stability and the drug release rate was slower. So, it is urgent to develop new DDSs with improved drug delivery efficiency.

In recent years, an increasing amount of attention has been paid to the environmentally responsive nanocarriers with improved drug release behavior, which can respond to the changes of the intrinsic biological signal, such as temperature, pH, enzyme, and ionic strength [23,24,25]. Among these changes, the redox potential gradient that exists between extracellular and intracellular environments is commonly used to design efficient DDSs based on functional PUs. Disulfide bonds are stable in body fluids and extracellular media because the concentration of glutathione (GSH) in body fluid and extracellular media is relatively low (less than 1 mM), whereas the high concentration of GSH (2–10 mM) in the diseased cells can break the disulfide bond. Guan et al. studied the location of disulfide bonds based on the Pus’ main chain that could optimize the release characteristics of PU-based nanocarriers. A rapid redox stimuli release behavior was observed on the PU-based nanocarriers whose disulfide bonds were mainly located between the hydrophobic core and the hydrophilic shell [26].

Despite their ease of synthesis, PUs that are fully degraded in response to an intracellular reducing environment have been the subject of fewer studies about controlled drug delivery [17]. Hence, the amphiphilic degradable polymers with disulfide bonds in the main chain are of great interest, whose advantage is that the disulfide bonds exhibit a reduction sensitivity in the intracellular reduction environment. The nanocarriers rapidly peel off the hydrophilic shell, leading to instability of the carrier. Finally, the nanocarriers accelerate disintegration and release the drug more quickly and efficiently [27,28].

**Scheme 1:**
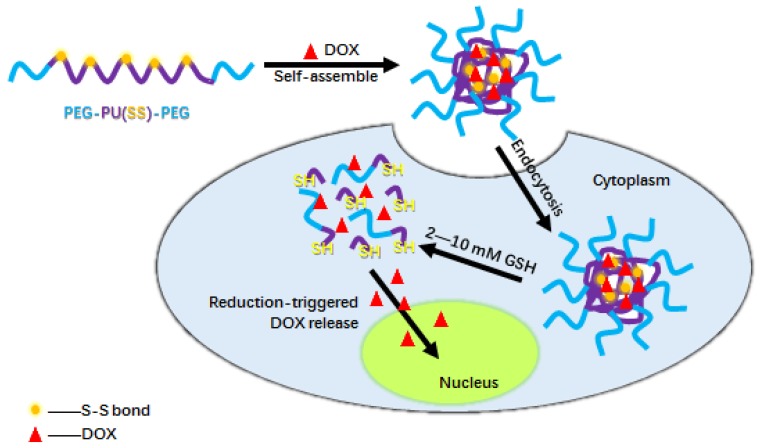
The illustration of the preparation of DOX-loaded PEG-PU(SS)-PEG micelles for reduction-responsive intracellular drug release.

In this paper, a new type of amphiphilic triblock polymer, polyethylene glycol -polyurethane-polyethylene glycol polymer material (PEG-PU(SS)-PEG) was synthesized and corresponding micelles were prepared for redox-triggered intracellular delivery of lipophilic anticancer drugs DOX (Scheme 1). PEG-PU(SS)-PEG copolymers could be readily prepared from condensation reaction between LDI and a disulfide-containing polylactide diol (PLA-SS-PLA) followed by hydroxyl terminated polyethylene glycol (PEG-OH) at both ends. The size distribution, zeta potential, stability, and drug release properties were fully investigated. The release behavior of DOX-loaded PEG-PU(SS)-PEG micelles in C6 cells was studied by fluorescence microscopy. In addition, the antitumor activity of the reduction-sensitive DOX-loaded PEG-PU(SS)-PEG micelles on C6 cells was investigated by CCK-8 assays.

## 2. Experimental Section

### 2.1. Materials

2,2’-Dithiodiethanol (HES) was purchased from Alfa Aesar (Shanghai, China), dried over CaH_2_, and purified by vacuum distillation. *N*,*N*-dimethyl formamide (DMF) was dried by refluxing over CaH_2_ and distilled under dry nitrogen. Toluene was dried by refluxing over Na and distilled under dry nitrogen. Tin octoate (Sn(Oct)_2_), LDI, and ethyl acetate (EAC) were purchased from Aladdin Industrial Co., Ltd. (Shanghai, China). Dithiothreitol (DTT) and 2-(2-methoxy-4-nitrophenyl)-3-(4-nitrophenyl)-5-(2,4-disulfonic acid benzene)-2*H*-tetrazole monosodium salt (CCK-8) was purchased from Dongren Chemical Technology Co., Ltd. (Suzhou, China). DOX was obtained from Beijing Zhongshuo Pharmaceutical Technology Development Co., Ltd. (Beijing, China). All other chemicals were of analytical grade and used without further purification. PLA-SS-PLA and PLA diols were purchased from Jinan Daigang Biomaterial Co., Ltd. (Jinan, China).

### 2.2. Characterization

^1^H NMR spectra were recorded on a Bruker 400 MHz (Bruker, Billerica, MA, USA) resonance spectrometer. The molecular weight and molecular weight distribution of the polymer were determined by PL GPC50 gel permeation chromatography (GPC). The GPC was equipped with 10E4, 2M Jordi (Agilent, Santa Barbara, CA, USA) gel column (DMF was used as eluent and the flow rate was 1.0 mL/min). The particle size and zeta potential of micelles were measured with a Zetasizer Nano-ZS dynamic light-scattering (DLS) instrument (Malvern, Malvern, UK). The morphology of micelles was observed with scanning electron microscope (SEM) SU-8010 (Hitachi, Tokyo, Japan). The fluorescence intensity of DOX was detected by an F-4600 (Hitachi, Tokyo, Japan) fluorescence spectrophotometer at 25 °C. CCK-8 experimental data were measured by a Synergy H4 mixed multifunction enzyme scale instrument (BioTek, Winooski, VT, USA).

### 2.3. Synthesis of PEG-PU(SS)-PEG and PEG-PU-PEG

**Scheme 2:**
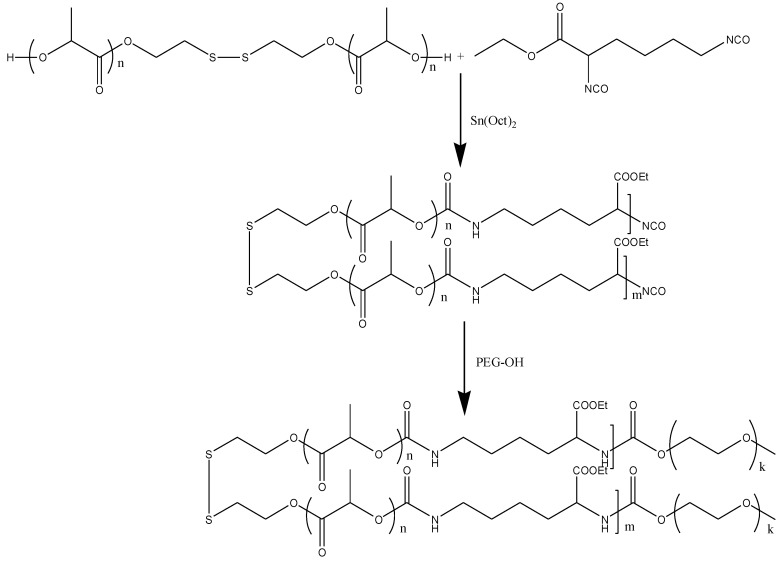
The synthesis and structure of PEG-PU(SS)-PEG.

Under the nitrogen protection, LDI (0.119 g, 0.525 mmol), PLA-SS-PLA (0.50 g, 0.5 mmol), 7.6 mL DMF, and 6 mg Sn(Oct)_2_ were added sequentially to the reactor and reacted at 65 °C for 48 h. After cooling to room temperature, PEG-OH (0.25 g, 0.025 mmol) was added dropwise to the above solution and the reaction was continued at 25 °C for 48 h. Then the reaction solution was dripped into ice-ethyl ether for sedimentation. The polymer was centrifuged and dried for 48 h (PEG-OH was treated with toluene to remove water; toluene and DMF were re-distilled before the experiment). Yield: 82%. The specific synthetic route is shown in Scheme 2.

The synthesis procedure of PEG-PU-PEG is basically the same as PEG-PU(SS)-PEG, except that PLA-SS-PLA diol is replaced by PLA diol.

### 2.4. Preparation and Characterization of PEG-PU(SS)-PEG Micelles

PEG-PU(SS)-PEG micelles were prepared via a dialysis method. The specific procedure was as follows: 2 mg of PEG-PU(SS)-PEG was dissolved in 1 mL of dimethyl sulfoxide (DMSO) under stirring at room temperature for 12 h. Deionized water (1.5 mL) was slowly added dropwise to the DMSO solution of PEG-PU(SS)-PEG. After the addition was completed, the prepared micellar solution was loaded into a pre-prepared dialysis bag (SPECTRA/POR, MWCO: 3500, (Spectrum Labs, Rancho Dominguez, CA, USA) and dialyzed against PB (10 mM, pH 7.4) for 24 h.

The particle size and distribution of the PEG-PU(SS)-PEG micelles were determined by DLS. Micellar solutions were filtered using a 0.45 μm filter before testing. The morphology of the PEG-PU(SS)-PEG micelles was observed by scanning electron microscope (SEM). Sample Preparation: The micellar solution was diluted to 0.1 mg/mL. A 10 μL portion of micellar solution was dropped on the silicon pellet and dried at room temperature.

In this study, the critical micelle concentration (CMC) of PEG-PU(SS)-PEG micelles was determined by using pyrene as a fluorescence probe. Thirteen kinds of micellar solution with a different concentration gradient were prepared. Then, a 30 μL portion of pyrene-acetone solution (1.622 × 10^−5^ g/mL) was added under dark conditions. The excitation wavelength of the spectrofluorometer was set to 330 nm and the fluorescence intensity of the micellar solution was measured in the scan range from 350 to 500 nm.

### 2.5. Degradation of PEG-PU(SS)-PEG Micelles under Reducing Conditions

The degradation of the PEG-PU(SS)-PEG micelles under reducing conditions (10 mM DTT) was followed by using DLS to measure the particle size of the micelles. In brief, two micellar solutions (2 mL) were prepared, one of which was bubbled under nitrogen to remove the oxygen. The well-dosed DTT was added to a glass cell of PEG-PU(SS)-PEG micelles (0.1 mg/mL) under nitrogen protection. PEG-PU(SS)-PEG micelles without DTT were used as a control. The two glass sample cells were then sealed with rubber stoppers, shaken evenly, and placed in a 37 °C shaker (200 rpm). The DLS was measured to track the change in particle size of the micelles at selected time points.

### 2.6. Preparation of Encapsulated-DOX PEG-PU(SS)-PEG Micelles and Determination of Drug Loading

In this experiment, the common anticancer drug doxorubicin (DOX) was chosen as a model drug. Since it was a fluorescent-sensitive substance, it was necessary to protect it from light during operation.

According to the theoretical drug loading (10% or 20%), the required DOX was dissolved in the DMSO solution of PEG-PU(SS)-PEG and sonicated for 30 min. Then, 1.5 mL of water was added dropwise into the above solution under vigorous stirring and the solution was sonicated again for 1 h. The mixed solution was then transferred into a dialysis bag (SPECTRA/POR, MWCO: 3500) and dialyzed against PB (10 mM, pH 7.4) for 24 h. The entire experiment was processed in dark conditions.

In order to determine the content of DOX, a certain amount of lyophilized drug-loaded micelles was dissolved in DMSO and sonicated for 1 h. The fluorescence intensity was measured with an F-4600 fluorescence spectrophotometer, wherein the corresponding drug loading content (DLC) and drug loading efficiency (DLE) of DOX were calculated according to the fluorescence standard curve. DLC and DLE were calculated based on Equations (1) and (2), respectively.

DLC (%) = weight of loaded drug/total weight of loaded drug and polymer × 100
(1)

DLE (%) = weight of loaded drug/weight of drug in feed × 100
(2)

### 2.7. In Vitro Drug Release of Drug-Loaded Micelles

The in vitro release of DOX from PEG-PU(SS)-PEG micelles was investigated at 37 °C in a constant temperature shaker (200 rpm). PB solution (10 mM, pH 7.4) containing 10 mM DTT and PB solution were selected for comparison. Drug-released samples were divided into three groups: (a) release of reduction-sensitive PEG-PU(SS)-PEG micelles in PB (10 mM, pH 7.4); (b) release of reduction-sensitive PEG-PU(SS)-PEG micelles in PB containing 10 mM DTT (10 mM, pH 7.4); (c) release of non-reduction-sensitive PEG-PU-PEG micelles in PB containing 10 mM DTT (10 mM, pH 7.4). DOX-loaded micelles were prepared in three dialysis bags (MWCO = 12,000–14,000) and immersed into 20 mL of the appropriate media. At the scheduled time, 4 mL of release media was removed and replenished with an equal volume of fresh release media. After 24 h, the sampling ended and the fluorescence of DOX was determined by an F-4600 fluorescence spectrophotometer to determine the content of drug released at each time point. Each release experiment was run in parallel three times.

### 2.8. CCK-8 Assays

The cytotoxicity of PEG-PU(SS)-PEG micelles was evaluated using C6 cells. C6 cells were cultivated in RPMI 1640 media supplemented with 10% fetal bovine serum (FBS), 1% l-glutamic acid, antibiotics penicillin (100 IU/mL), and streptomycin (100 μg/mL) at 37 °C in a humidified atmosphere of 5% CO_2_. C6 cells were digested when they were cultured to about 80% of the culture flask and then seeded in the 96-well plates (1 × 10^4^ cells/well). This experiment had five groups of test groups (micellar concentrations of 0.2, 0.4, 0.6, 0.8, and 1 mg/mL), a control group, and a blank group. After incubation for 24 h, the medium was sucked away and replaced by 100 μL of fresh medium containing various concentrations of micelle for another 24 h. Afterward, a 10 μL portion of CCK-8 solution was added to each well for 0.5–4 h of incubation. The absorbance (A) of each well was measured using a Synergy Model H4 hybrid multi-plate reader, and the blank medium well was used as a zero group for zero adjustment. The experiment is performed in multiples. According to the plate reader results, cell viability was calculated based on Equation (3):

Cell viability (%) = (A_T_ − A_B_)/(A_C_ − A_B_) × 100 (3)

In the formula:

A_T_: Absorbance of test group
A_C_: Absorbance of control group
A_B_: Absorbance of blank group

As described above, the antitumor activity of DOX-loaded PEG-PU(SS)-PEG micelles and free DOX·HCl was also studied by CCK-8 assays. C6 cells were cultivated in RPMI 1640 media supplemented with 10% FBS, 1% l-glutamic acid, antibiotics penicillin (100 IU/mL), and streptomycin (100 μg/mL) at 37 °C in a humidified atmosphere of 5% CO_2_. C6 cells were digested when they were cultured to about 80% of the culture flask and then seeded in 96-well plates (5 × 10^3^ cells/well). This experiment had seven groups of test groups (DOX concentrations of 0.625, 1.25, 2.5, 5, 10, 20, and 40 μg/mL), a control group, and a blank group. After incubation for 24 h, the medium was sucked away and replaced by 100 μL of fresh medium containing various concentrations of micelle for 48 h. Afterward, a 10 μL portion of CCK-8 solution was added to each well for 0.5–4 h of incubation. The absorbance (A) of each well was measured using a Synergy H4 hybrid multi-plate reader, and the blank medium well was used as a zero group for zero adjustment. The experiment is performed in multiples. According to the plate reader results, the cell viabilities were calculated as described above.

### 2.9. Cell Internalization

The cellular uptake and intracellular drug release behaviors of DOX-loaded PEG-PU(SS)-PEG micelles were examined in C6 cells using a fluorescence microscope. C6 cells were cultivated in RPMI 1640 media supplemented with 10% FBS, 1% l-glutamic acid, antibiotics penicillin (100 IU/mL), and streptomycin (100 μg/mL) at 37 °C in a humidified atmosphere of 5% CO_2_. C6 cells were digested when they were cultured to about 80% of the culture flask and then seeded in 24-well plates (6 × 10^4^ cells/well). After incubation for 24 h, the medium was sucked away and replaced by fresh medium containing DOX-loaded PEG-PU(SS)-PEG micelles, DOX-loaded PEG-PU-PEG micelles, or DOX·HCl (final DOX concentration of 40 μg/mL) for 0.5 h, 2 h, and 4 h. The medium in each well was removed and washed three times with phosphate buffered saline (PBS, 10 mM, pH 7.4). Thereafter, the cells were fixed with 1 mL of 4% paraformaldehyde for 30 min. After removal of the paraformaldehyde, the cells were rinsed with PBS another three times, and stained with 4,6-diamidino-2-phenylindole (DAPI) for 15 min. Finally, the excess staining solution was washed three times with PBS and fluorescence images of cells were obtained with a fluorescence microscope.

### 2.10. Flow Cytometry Measurements

The cellular uptake and intracellular drug release behaviors of DOX-loaded PEG-PU(SS)-PEG micelles were also examined in C6 cells using flow cytometry measurements. C6 cells were seeded in 6-well plates (5 × 10^5^ cells/well) and incubated for 24 h. Then, the medium was sucked away and replaced by fresh medium containing DOX-loaded PEG-PU(SS)-PEG micelles, DOX-loaded PEG-PU-PEG micelles, or DOX·HCl (final DOX concentration of 20 μg/mL) for 0.5 h, 2 h, and 4 h. Cells in each well were digested with 0.25% trypsin and collected in centrifuge tubes. The suspensions were centrifuged at 0.5 *g* for 3 min at 4 °C. Afterwards, the precipitate was washed three times with PBS, and re-suspended in 1 mL of PBS. Fluorescence intensity in cells was measured by Accuri C6 flow cytometry. The intracellular fluorescence histograms could be obtained by BD Accuri C6 software (264.21). The data were analyzed from the fluorescence data of 20,000 cells.

## 3. Results and Discussion

### 3.1. Synthesis of Reduction-Sensitive Degradable PEG-PU(SS)-PEG Triblock Copolymer

**Figure 1:**
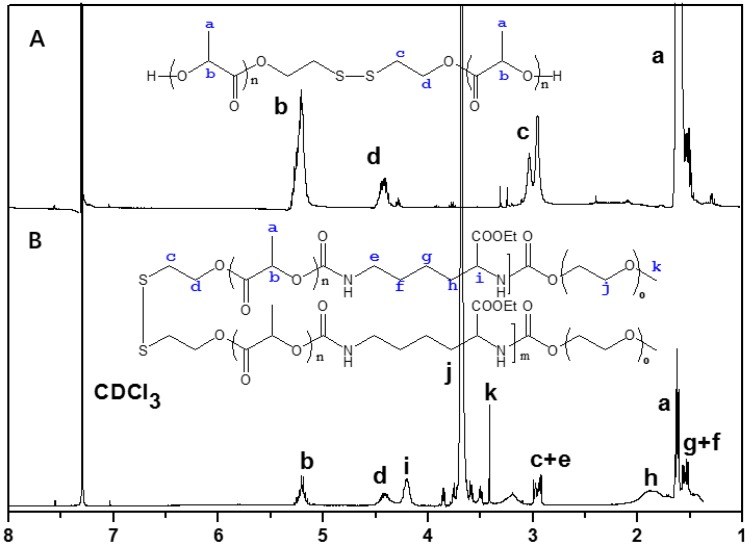
^1^H NMR spectrum (400 MHz, CDCl_3_) of (**A**) PLA-SS-PLA diol and (**B**) PEG-PU(SS)-PEG.

PEG-PU(SS)-PEG was synthesized via condensation reaction of polylactide diol (PLA-SS-PLA) and diisocyanate (LDI), then reacted with PEG-OH. The polymer was characterized by ^1^H NMR and GPC. The ^1^H NMR spectrum of PEG-PU(SS)-PEG polymers showed that the peaks at chemical shift *δ* 1.67 and *δ* 5.20 corresponded to the hydrogen of methyl and methyne of PLA-SS-PLA, respectively (Figure 1). The peak at *δ* 2.95 corresponded to methylene hydrogen of PLA-SS-PLA. The chemical shift of 3.66 is the signal of methylene hydrogen in the PEG backbone, and *δ* 1.24 and *δ* 1.97 are signals for methylene hydrogen in LDI. GPC data (Table 1) showed that PEG-PU(SS)-PEG had a single peak and a narrower molecular weight distribution (the Polymer dispersity index (PDI) value was 1.03). All the results demonstrated the successful synthesis of PEG-PU(SS)-PEG in this experiment. In addition, PEG-PU-PEG without disulfide bonds in the backbone was synthesized as a control.

### 3.2. Formation and Characterization of PEG-PU(SS)-PEG Micelles

**Figure 2:**
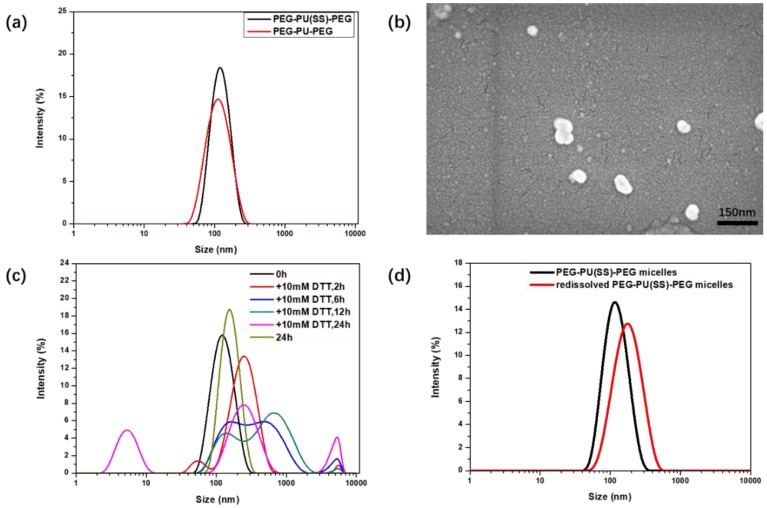
(**a**) Size and size distribution of PEG-PU(SS)-PEG micelles and PEG-PU-PEG micelles. (**b**) SEM image of PEG-PU(SS)-PEG micelles. (**c**) Change of size distribution of PEG-PU(SS)-PEG micelles at 37 °C, 10 mM DTT solution. (**d**) Changes in particle size of redissolved DOX-loaded PEG-PU(SS)-PEG micelles after freeze-drying.

In this study, PEG-PU(SS)-PEG micelles were prepared via a dialysis method. The size of the PEG-PU(SS)-PEG micelles was 116.7 nm measured by DLS at 25 °C, which indicated that the PEG-PU(SS)-PEG micelles had a small size and uniform distribution and reached a monodisperse state (Figure 2a). As shown in Figure 2b, SEM revealed that the micelles were individual particles with great dispersion and regular spherical shape, which was in line with those obtained from DLS. As the previous work demonstrated [29], such small size and a homogeneous distribution are helpful to the cell internalization of PU micelles. The CMC of PEG-PU(SS)-PEG micelles was determined by a fluorescence method using pyrene as a probe. As shown in Table 2, PEG-PU(SS)-PEG micelles had a lower CMC value of 1.6 mg/L than that of PEG-PU-PEG, which might be related to the difference in molecular weight of the hydrophobic block, as demonstrated in some references [30,31]. Due to the low CMC, the micelles are more stable in water and capable of long circulation in vivo, which is advantageous for the drug carrier to accumulate at the tumor site through enhanced permeability and retention (EPR) effects. The stability of the micelle can also be demonstrated by the good redissolving status of the freeze-drying PEG-PU(SS)-PEG micelle.

### 3.3. Reduction-Responsive Size Change of PEG-PU(SS)-PEG Micelles

It is well-known that disulfide bonds are easily broken down into thiol under reducing conditions. In this work, in order to investigate the potential intracellular degradation of PEG-PU(SS)-PEG micelles, the change of particle size and size distribution in reducing environment were monitored by DLS. It is worth noting that in the presence of 10 mM DTT, the micelles underwent rapid and significant swelling and the average particle size increased. As shown in Figure 2c, the size distribution of PEG-PU(SS)-PEG micelles after incubation for 6 h with 10 mM DTT increased significantly, with the average particle size increasing from 101.3 to 254.8 nm, while the PDI also increased from 0.15 to 0.463. After 24 h, the micelles clearly broke, DLS showed that it divided into many peaks, and some aggregates appeared. In contrast, the size of the micelles did not change much in the non-reducing environment. It was likely that the disulfide bonds between the hydrophilic shell and the hydrophobic core of the PEG-PU(SS)-PEG micelles were reductively degraded. The hydrophilic shells fell off, and the micelles swelled, resulting in instability or aggregation of the PEG-PU(SS)-PEG micelles. Degradation experiments showed that PEG-PU(SS)-PEG micelles were responsive to the reducing environment and could break rapidly under reducing conditions that simulate the nucleus and cytoplasm.

### 3.4. Loading and In Vitro Release of DOX

To evaluate the loading capacity and the in vitro release capacity of PEG-PU(SS)-PEG micelles as a drug carrier, DOX was used as a model drug. DOX is one of the most potent chemotherapeutic agents, and its principle of action is to insert itself into DNA and thus inhibit the synthesis of nucleic acids [32,33].

DOX-loaded PEG-PU(SS)-PEG micelles were prepared via a dialysis method, and DOX-loaded PEG-PU–PEG micelles was used as a control. It was found that PEG-PU(SS)-PEG micelles could encapsulate DOX efficiently. Table 3 indicates that the encapsulation efficiency of PEG-PU(SS)-PEG micelles was slightly higher than that of PEG-PU-PEG micelles, which was approximately 65%. The size of DOX-loaded PEG-PU-PEG micelles had no appreciable change, which was approximately 116.2–144.6 nm. These data demonstrated that drug-loaded micelles were easily phagocytosed by the cells, thereby being beneficial for transporting DOX into the cells. The fluorescence of drug-loaded micelles was tested by a fluorescent spectrophotometer, and then the DLC and DLE were calculated according to the standard curve of DOX.

To further prove the stability of PEG-PU(SS)-PEG micelles and explore the probability for future clinical treatment, reconstitution experiments were performed on freeze-dried samples. First, a sample of 10% DOX-loaded PEG-PU-PEG micelles was freeze-dried for 24 h, then an equal volume of water was added to redissolve the sample, and the size of the micelles was measured again using DLS. The size of the redissolved micelles was 159.72 ± 0.54 nm, and the PDI was 0.142 ± 0.06 (Figure 2d). Compared with the micelles before freeze-drying, the size of the redissolved micelles changed slightly, further indicating that DOX-loaded PEG-PU-PEG micelles had a good physical stability, which was conducive to its use as a drug carrier.

**Figure 3:**
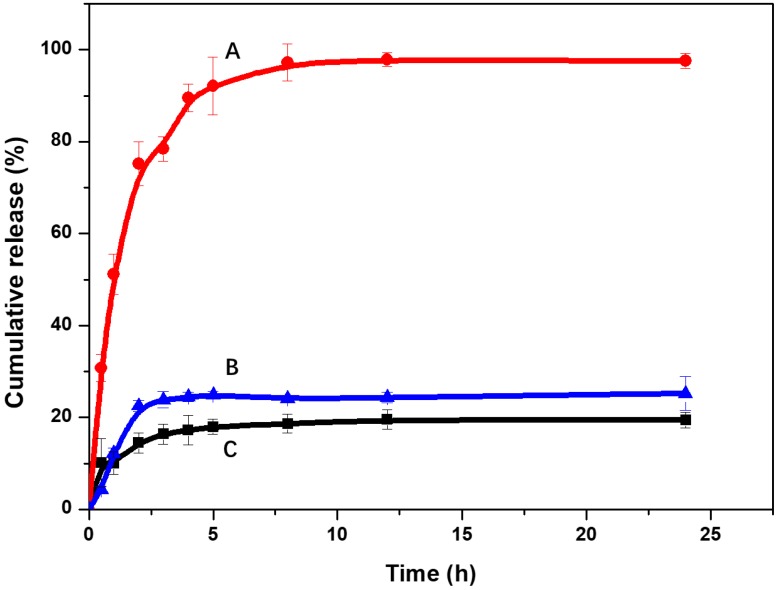
In vitro DOX release profile of DOX-loaded PEG-PU(SS)-PEG micelles at 37 °C (A: PEG-PU(SS)-PEG micelles + 10 mM DTT; B: PEG-PU-PEG micelles + 10 mM DTT; C: PEG-PU(SS)-PEG micelles). Error bars represent standard deviation (SD) for *n* = 3.

In vitro drug release experiments of drug-loaded micelles were carried out in a constant temperature shaker (200 rpm, 37 °C). As shown in Figure 3, owing to the reduction-degradable character of disulfide bonds in the backbone, approximately 75.25% of DOX was released within 2 h from drug-loaded PEG-PU(SS)-PEG micelles, and approximately 97.54% of DOX was released within 24 h from drug-loaded PEG-PU(SS)-PEG micelles. In contrast, only 25.2% of DOX was released within 24 h from reduction insensitive PEG-PU-PEG micelles under the same conditions. The results of the release experiment fully demonstrated that DOX-loaded PEG-PU(SS)-PEG micelles had reduction sensitivity and could release DOX rapidly under reducing conditions. The release results were consistent with the previous results that the micelles swell under reducing conditions of 10 mM DTT in the degradation reaction of PEG-PU(SS)-PEG micelles.

### 3.5. Cellular Uptake

**Figure 4:**
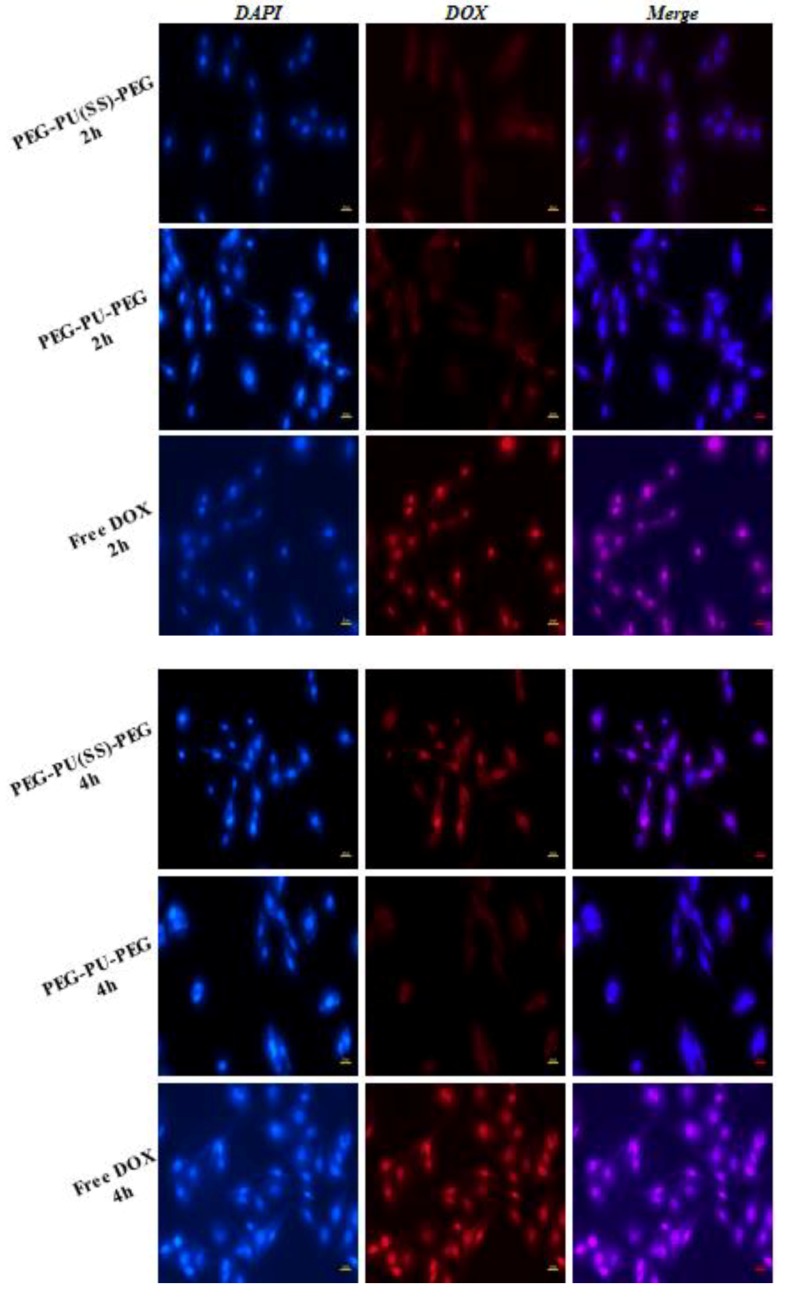
Fluorescence microscope images of C6 cells incubated with DOX-loaded PEG-PU(SS)-PEG micelles, DOX-loaded PEG-PU-PEG micelles, and free DOX· HCl for 2 h and 4 h (dosage: 25 μg DOX equiv/mL). For each panel, images from left to right showed cell nuclei stained by DAPI (blue), DOX fluorescence in cells (red), and overlays of two images. The scale bars correspond to 20 μm in all images.

Fluorescence microscopes were widely used in the study of intracellular drug delivery and release behavior. Taking DOX-loaded PEG-PU(SS)-PEG micelles as an example, the cellular uptake and intracellular release of DOX-loaded PEG-PU(SS)-PEG micelles in C6 cells were observed by fluorescence microscope. The experiment was divided into three groups: DOX-loaded PEG-PU(SS)-PEG micelles, DOX-loaded PEG-PU-PEG micelles, and free DOX·HCl, with three replicates in each group. As shown in Figure 4, C6 cells following 2 h incubation with DOX-loaded PEG-PU(SS)-PEG micelles showed strong red fluorescence of DOX in the cytoplasm. In contrast, DOX-loaded PEG-PU-PEG micelles showed only weak red fluorescence of DOX. Furthermore, the fluorescence intensity of DOX-loaded PEG-PU(SS)-PEG micelles incubated with C6 cells for 4 h significantly increased in the nucleus and cytoplasm. However, DOX-loaded PEG-PU-PEG micelles incubated with C6 cells for 4 h only showed some DOX fluorescence in the cytoplasm, and almost no red fluorescence appeared in the nucleus. In line with CCK-8 assays, free DOX was delivered more quickly into the nuclei of the C6 cells. In addition, the average fluorescence intensity of the reduction-sensitive PEG-PU(SS)-PEG micelles was obviously higher than that of the non-sensitive PEG-PU-PEG micelles at all intervals. The above experimental results showed that PEG-PU(SS)-PEG micelles could respond to the intracellular reducing environment and the disulfide bonds cleavage would lead to the disintegration of PEG-PU(SS)-PEG micelles and accelerate the release of DOX in tumor cells.

**Figure 5:**
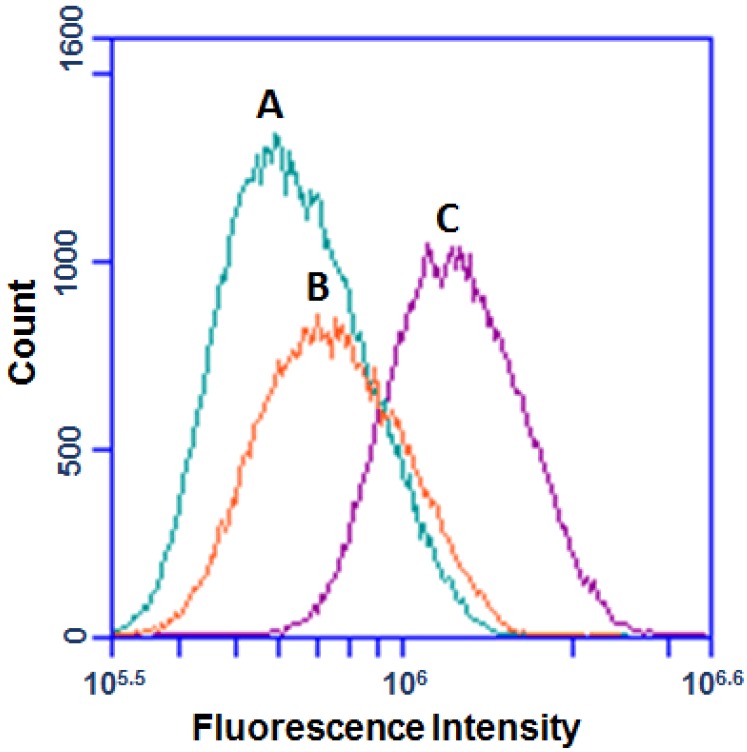
Flow cytometry measurements of cellular DOX level in C6 cells following 4 h incubation with (**A**) DOX-loaded PEG-PU-PEG micelles, (**B**) DOX-loaded PEG-PU(SS)-PEG micelles, and (**C**) free DOX·HCl. (DOX dosage: 30 μg/mL, cell counts were 10,000)

Flow cytometry assays are widely used to quantitatively determine the cell endocytosis and uptake of DOX-loaded micelles and FITC (fluoresceine isothiocyanate)-labeled micelles [34,35,36]. It has been reported that only the fluorescence of DOX released by the self-disassembling of DOX-loaded nanocarriers could be observed and measured by flow cytometry [34]. Therefore, the intensity of intracellular fluorescence was directly related to the amount of DOX released from the cells. As shown in Figure 5, the fluorescence intensity of C6 cells following 4 h incubation with DOX-loaded PEG-PU(SS)-PEG micelles was far greater than that of C6 cells following 4 h incubation with DOX-loaded PEG-PU-PEG micelles. In other words, DOX-loaded PEG-PU(SS)-PEG micelles were responsive to the reducing environment in tumor cells and released doxorubicin more quickly and more completely. The results of the flow cytometry assay were also consistent with the results measured by fluorescence microscope previously.

### 3.6. Cell Viability Analysis of DOX-Loaded PEG-PU(SS)-PEG Micelles

**Figure 6:**
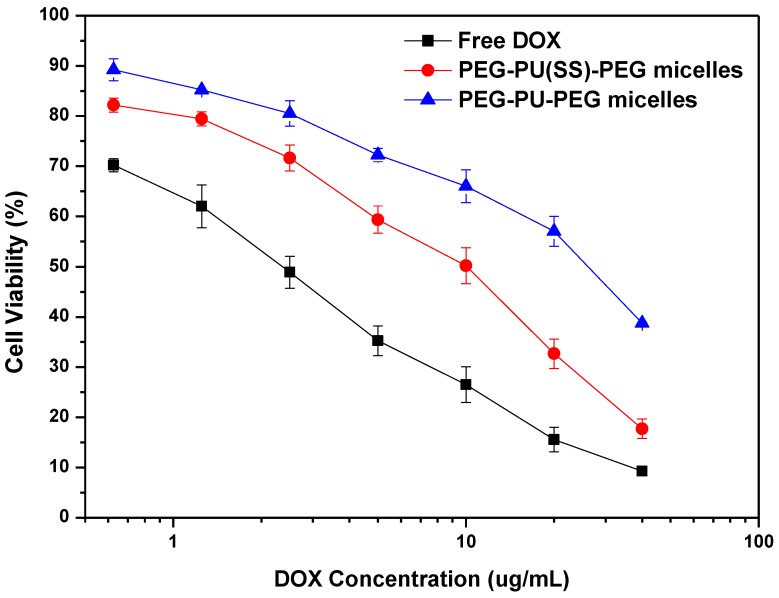
Anti-tumor activity of DOX-loaded PEG-PU(SS)-PEG micelles, DOX-loaded PEG-PU-PEG micelles, and free DOX as a function of DOX dosages. The cells (C6) were incubated with DOX-loaded micelles or free DOX for 48 h. Data are presented as the average ± standard deviation (*n* = 6).

**Figure 7:**
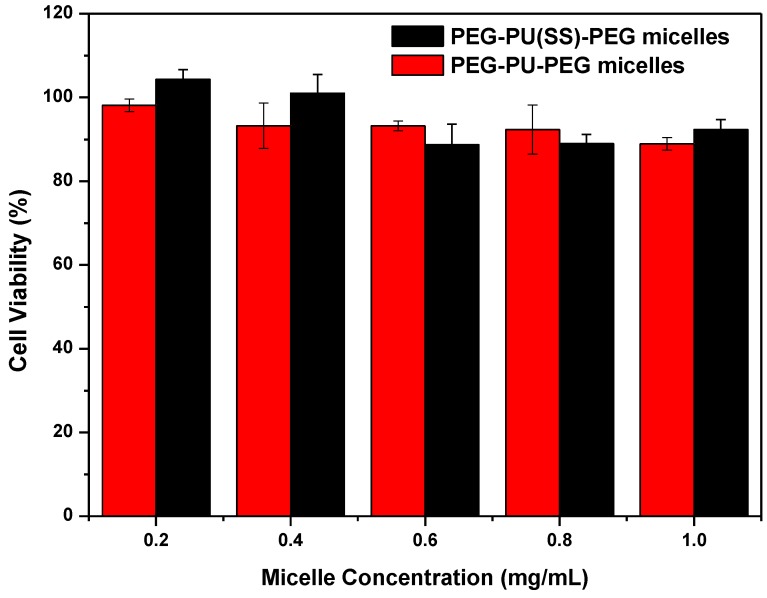
Cytotoxicity of PEG-PU(SS)-PEG and PEG-PU-PEG micelles. The cells (C6) were incubated with micelles for 24 h. Data are presented as the average ± standard deviation (*n* = 6).

The cytotoxicity and antitumor activity of reduction-responsive PEG-PU(SS)-PEG micelles were studied by CCK-8 assays. C6 cells were incubated with DOX-loaded PEG-PU(SS)-PEG micelles for 24 h at different DOX concentrations (0.625–40 μg/mL), and DOX-loaded PEG-PU-PEG micelles and free DOX·HCl were used as controls. DOX-loaded PEG-PU(SS)-PEG micelles showed significant dose-dependent antitumor activity compared to their counterparts, and led to more efficient delivery and release of DOX to the cytoplasm and nucleus. The IC_50_ was 7.02 μg /mL (DOX equivalents), lower than DOX-loaded PEG-PU-PEG micelles (25.68 μg/mL) (Figure 6). The reason is that the cellular entry enhancement and intracellular drug release can rapidly increase the concentration of drugs in the tumor cells, effectively killing cancer cells [37]. Under the same conditions, the antitumor activity of free DOX·HCl to C6 cells was slightly higher than that of other two formulations. This is because free DOX·HCl is a small molecule that directly enters the nucleus by passive diffusion, whereas DOX-loaded PEG-PU(SS)-PEG micelles and DOX-loaded PEG-PU-PEG micelles enter the cells to release DOX through endocytosis. In the biocompatibility experiment, the blank PEG-PU(SS)-PEG micelles did not show any cytotoxicity at a concentration up to 1 mg/mL, and the cells still had a viability of over 90% (Figure 7). These results demonstrated that reduction-responsive PEG-PU(SS)-PEG micelles had good biocompatibility and are likely to be used as pharmaceutical nanovehicles. In fact, the PU micelles based on the hydrophilic segment PEG and the biodegradable hydrophobic segments with excellent biocompatibility have been widely used in drug carriers, tissue engineering, and other biomedical fields [17,18,19,20].

## 4. Conclusions

In this work, biodegradable amphiphilic polymers PEG-PU(SS)-PEG with disulfide bonds in the main chain were successfully synthesized. In aqueous solution, the resulting PEG-PU(SS)-PEG polymers could self-assemble into micelles and load DOX to kill glioma cells with high expression. The new polymer micelles have the following advantages and highlights: (1) The micelles have good biocompatibility and hydrophobic segment biodegradability. (2) The micelles have small particle sizes and are favorable for drug delivery. (3) The micelles are stable under normal physiological conditions and can be circulated in human body fluid for a long time. (4) The micelles have a reduction responsiveness and can rapidly release the loaded drug DOX. On one hand, in vitro drug release experiments showed that the micelles exhibited excellent colloidal stability in the extracellular environment, which avoided the early release of drugs due to the dissociation of micelles. On the other hand, the micelles showed obvious reduction responsiveness after they had been endocytosed into cancer cells. Under the influence of the reducing substance GSH, the disulfide bond in the main chain broke, triggered the release of the loaded drug, and achieved the high release of the DOX. Cell experiments confirmed that treatment with DOX-loaded PEG-PU(SS)-PEG micelles significantly inhibited C6 cell growth compared to other groups. The above results indicate that PEG-PU(SS)-PEG micelles have good biocompatibility, stability, and reduction responsibility, and it is expected they will be used as a promising anticancer drug carrier for cancer treatment in the future.

## Figures and Tables

**Table 1:** Molecular characteristics of PEG-PU(SS)-PEG and PEG-PU-PEG.

Copolymer	*Mn*^a^ (^1^H NMR)	*Mn*^b^ (GPC)	PDI ^b^ (GPC)
PEG-PU(SS)-PEG	15,000	28,400	1.03
PEG-PU-PEG	18,800	31,400	1.02

^a^ Measured by ^1^H NMR (400 MHz, CDCl_3_); ^b^ Determined by GPC (eluent: DMF, flow rate: 1 mL/min, standard: 30 °C).

**Table 2:** Characterization of PEG-PU(SS)-PEG micelles and PEG-PU-PEG micelles.

Block Copolymers	Micelle Size (nm) ^a^	PDI	Zeta Potential (mv) ^a^	CMC (mg/L) ^b^
PEG-PU(SS)-PEG	116.7 ± 1.9	0.08 ± 0.03	−20.8	1.64
PEG-PU-PEG	139.8 ± 1.1	0.05 ± 0.01	−26.6	5.62

^a^ Measured at 25 °C using a Malvern dynamic light scattering instrument. ^b^ Determined using the pyrene fluorescent probe method.

**Table 3:** Characteristics of DOX-loaded PEG-PU(SS)-PEG micelles and PEG-PU-PEG micelles.

Sample	Theoretical Drug Loading Content (wt %)	DLC ^a^ (wt %)	DLE ^a^ (%)	Micelle Size ^b^ (nm)	PDI ^b^
PEG-PU(SS)-PEG	10	6.59	65.89	116.2 ± 2.8	0.20 ± 0.01
	20	13.13	65.67	144.0 ± 0.9	0.15 ± 0.02
	10	6.07	60.72	141.4 ± 1.4	0.16 ± 0.02
PEG-PU-PEG	20	12.54	62.70	154.9 ± 1.6	0.17 ± 0.01

^a^ DLC and DLE was determined by fluorescence measurements. ^b^ Measured at 25 °C using a Malvern dynamic light scattering instrument.
